# Low triiodothyronine levels correlate with high B-type natriuretic peptide levels in patients with heart failure

**DOI:** 10.1038/s41598-021-01454-5

**Published:** 2021-11-08

**Authors:** Hirotake Takahashi, Yusuke Kashiwagi, Tomohisa Nagoshi, Yoshiro Tanaka, Yuhei Oi, Haruka Kimura, Kousuke Minai, Michihiro Yoshimura

**Affiliations:** Division of Cardiology, Department of Internal Medicine, The Jikei University School of Medicine, 3-25-8, Nishi-shimbashi, Minato-ku, Tokyo, 105-8461 Japan

**Keywords:** Cardiovascular diseases, Heart failure

## Abstract

Thyroid hormone metabolism can be closely associated with cardiovascular disorders. We examined the relationship between low triiodothyronine (T3) levels and heart failure status, including B-type natriuretic peptide (BNP) levels, in 625 patients with cardiovascular disorders who underwent cardiac catheterization. A multiple regression analysis revealed that the left ventricular ejection fraction (LVEF), hemoglobin (Hb) levels, sex (male), free T3 (FT3) levels, and estimated glomerular filtration rate (eGFR) were significantly negatively associated with the log BNP value, while age was significantly positively associated with the log BNP value (*P* < 0.001 each). Furthermore, the log BNP and age were significantly negatively associated with the FT3 levels, while the Hb and body mass index (BMI) were significantly positively associated with the FT3 levels (*P* < 0.001 each). Theoretically constructed structure equation modeling (SEM) revealed an inverse association between FT3 and BNP (β = −0.125, *P* = 0.002), and the same relationship remained in the patient group with normal-range BNP values (β = −0.198, *P* = 0.008). We demonstrated a significant relationship between high BNP and low serum FT3 levels, and this relationship remained significant in patients with normal BNP levels. These results indicate that low T3 is associated with high plasma BNP levels rather than worsening of hemodynamics.

## Introduction

The synthesis and secretion of thyroid hormone are largely regulated by the hypothalamus-pituitary-thyroid axis^1^. Under the regulation of thyroid-stimulating hormone (TSH), the thyroid gland synthesizes and secretes mainly thyroxine (T4) (approximately 85%) and a smaller percentage of 3,5,3’-triiodothyronine (T3). T4 is converted to T3, which is the active form of thyroid hormone, by 5'-monodeiodination in the liver, kidney, and skeletal muscle^2,3^. T3 and T4 also exert a negative feedback effect that suppresses the synthesis and secretion of TSH in the pituitary gland^2,4^.

T3 shows various biological effects systemically, including stimulation of tissue thermogenesis, skeletal development, modulation of the appetite and food intake, and regulation of the body weight^5–7^. The serum T3 concentration decreases under conditions of critical illness, such as severe systemic infection, trauma, and starvation^8–10^. This low T3 syndrome is a beneficial adaptation and protective response that decreases energy consumption under pathological conditions^11^.

In the heart, T3 plays an important role in modulating myocardial contractility and hemodynamics by regulating the expression of the cardiac gene encoding cardiac protein^12^. An altered thyroid hormone metabolism closely reflects the pathological condition of cardiovascular systems^13–16^. Indeed, a low T3 syndrome has been reported in patients with heart failure, and the magnitude of this decrease in T3 is related to the prognosis of cardiovascular disorders^17–22^. Furthermore, previous studies have reported that the serum T3 level was inversely associated with the level of B-type natriuretic peptide (BNP) or N-terminal pro BNP (NT-pro BNP), a biomarker of heart failure severity^23–26^. However, thyroid hormone reportedly stimulates the release of BNP from both cultured atrial and ventricular myocytes in a dose-dependent manner^27^. In addition, thyroid hormone may be involved in the process of left ventricle (LV) remodeling, even in euthyroid patients^28^. For these reasons, the relationship between BNP and T3 levels needs to be reconsidered.

In the present study, we investigated the relationship between BNP and free T3 (FT3) levels in patients with cardiovascular disorders, taking into account other clinical factors that have the potential to affect the BNP or FT3 levels.

## Results

### Patient characteristics

Table 1 shows the clinical characteristics of the patients in this study. The average FT3 and free T4 (FT4) levels were 2.33 ± 0.32 pg/mL and 1.25 ± 0.23 ng/dL, respectively. The median TSH level was 1.43 μIU/mL (interquartile range [IQR] 0.91–2.22 μIU/mL), the median BNP level was 36.0 pg/mL (IQR 15.9–96.3 pg/mL), and the median LV ejection fraction (LVEF) was 60.8% (IQR 50.6–66.1%).

**Table 1:** The clinical characteristics of the patients (n = 625).

Characteristics	Number (%), mean ± SD or median (25th, 75th percentile)
Male (n, %)	541 (86.6)
Age (years)	67 (58, 74)
BMI (kg/m^2^)	24.2 (22.0, 26.5)
SBP (mmHg)	134 (116, 150)
DBP (mmHg)	70 (62, 79)
Heart rate (beats per minutes)	73 (60, 84)
Hb (g/dL)	14.0 ± 1.74
eGFR (mL/min/1.73 m^2^)	71.0 (62.1, 83.7)
Total bilirubin (mg/dL)	0.8 (0.6, 1.0)
Albumin (g/dL)	3.94 ± 0.38
Fasting blood sugar (mg/dL)	105 (94, 125)
HbA1c (%)	6.1 (5.7, 6.7)
HDL (mg/dL)	49 (41, 60)
LDL (mg/dL)	93 (76, 113)
Triglyceride (mg/dL)	100 (74, 138)
FT3 (pg/mL)	2.33 ± 0.32
FT4 (ng/dL)	1.25 ± 0.23
TSH (μIU/mL)	1.43 (0.91, 2.22)
BNP (pg/mL)	36.0 (15.9, 96.3)
LVEF (%)	60.8 (50.6, 66.1)
LVESVI (mL/m^2^)	23.7 (17.9, 34.3)
LVEDVI (mL/m^2^)	61.8 (49.8, 74.4)
**Underlying main cardiovascular disease (n, %)**	
Ischemic heart disease	506 (81)
Cardiomyopathy	52 (8.3)
Valvular disease	18 (2.9)
Arrhythmia	10 (1.6)
Macrovascular disease	2 (0.3)
Congenital heart disease	1 (0.2)
Other diseases	36 (5.8)
**ACCF/AHA Stage of heart failure (n, %)**	
Stage A	17 (2.7)
Stage B	462 (73.9)
Stage C	142 (22.7)
Stage D	4 (0.6)
**Medications (n, %)**	
Antiplatelet agent	491 (78.6)
ACE inhibitor	185 (29.6)
ARB	245 (39.2)
Beta-blocker	320 (51.2)
Diuretics	149 (23.8)

SD, standard deviation; BMI, body mass index; SBP, systolic blood pressure; DBP, diastolic blood pressure; Hb, hemoglobin; eGFR, estimated glomerular filtration rate; HDL, high-density lipoprotein cholesterol; LDL, low-density lipoprotein cholesterol; FT3, free triiodothyronine; FT4, free thyroxine; TSH, thyroid-stimulating hormone; BNP, B-type natriuretic peptide; LVEF, left ventricular ejection fraction; LVESVI, left ventricular end-systolic volume index; LVEDVI, left ventricular end-diastolic volume index; ACCF, American College of Cardiology Foundation; AHA, American Heart Association; ACE, angiotensin-converting enzyme; ARB, Angiotensin II Receptor Blocker.

### The comparison of clinical data between groups separated by the median level of FT3 (2.33 pg/mL)

To investigate the relationship between the FT3 level and various clinical data, we divided all patients into 2 groups by the median level of FT3 (2.33 pg/mL) and compared the two groups. As shown in Table 2, the number of male patients was significantly smaller (*P* < 0.001), the age and TSH level were significantly higher (each *P* < 0.001), and the body mass index (BMI), diastolic blood pressure (DBP), hemoglobin (Hb) level, estimated glomerular filtration rate (eGFR), total bilirubin level, albumin level, and triglyceride level were significantly lower in the low-T3 group (FT3 ≤ 2.33 pg/mL) than in the high-T3 group (FT3 > 2.33 pg/mL) (total bilirubin level: *P* = 0.01, and the others: *P* < 0.001, respectively). Furthermore, although there was no marked difference between the groups with regard to the left ventricular ejection fraction (LVEF), left ventricular end-systolic volume index (LVESVI), or left ventricular end-diastolic volume index (LVEDVI), the BNP level was significantly higher in the low-T3 group than in the high-T3 group (*P* < 0.001).

**Table 2:** A comparison of the clinical data between groups separated by the median level of FT3 (2.33 pg/mL).

	FT3 ≤ 2.33 (pg/mL) (n = 314)	FT3 > 2.33 (pg/mL) (n = 311)	*P* value
Male (%)	80.6	92.6	< 0.001
Age (years)	70 (62, 76)	63 (56, 71)	< 0.001
BMI (kg/m^2^)	23.4 (21.5, 25.7)	24.9 (22.8, 27.4)	< 0.001
SBP (mmHg)	134 (118, 150)	134 (114, 150)	0.754
DBP (mmHg)	68 (61, 76)	72 (63, 81)	< 0.001
Heart rate (beats per minutes)	74 (61, 85)	71 (60, 83)	0.549
Hb (g/dL)	13.5 ± 1.84	14.4 ± 1.49	< 0.001
eGFR (mL/min/1.73 m^2^)	68.8 (59.5, 80.0)	74.4 (65.4, 88.5)	< 0.001
Total bilirubin (mg/dL)	0.8 (0.6, 0.9)	0.8 (0.6, 1.0)	0.010
Albumin (g/dL)	3.84 ± 0.42	4.03 ± 0.32	< 0.001
Fasting blood sugar (mg/dL)	105 (94, 126)	105 (94, 123)	0.404
HbA1c (%)	6.1 (5.7, 6.8)	6.0 (5.7, 6.7)	0.503
HDL (mg/dL)	52 (41, 62)	48 (41, 58)	0.051
LDL (mg/dL)	95 (76, 114)	92 (75, 110)	0.566
Triglyceride (mg/dL)	91 (66, 130)	110 (85, 147)	< 0.001
FT4 (ng/dL)	1.24 ± 0.24	1.27 ± 0.22	0.102
TSH (μIU/mL)	1.57 (0.98, 2.47)	1.29 (0.83, 2.02)	< 0.001
BNP (pg/mL)	52.1 (21.4, 139.7)	25.7 (12.0, 64.0)	< 0.001
LVEF (%)	60.1 (48.1, 66.1)	61.5 (52.9, 66.0)	0.093
LVESVI (mL/m^2^)	24.2 (18.2, 35.4)	23.3 (17.7, 32.6)	0.104
LVEDVI (mL/m^2^)	62.2 (50.9, 75.9)	61.2 (49.3, 73.9)	0.170

FT3, free triiodothyronine; BMI, body mass index; SBP, systolic blood pressure; DBP, diastolic blood pressure; Hb, hemoglobin; eGFR, estimated glomerular filtration rate; HDL, high-density lipoprotein cholesterol; LDL, low-density lipoprotein cholesterol; FT4, free thyroxine; TSH, thyroid-stimulating hormone; LVEF, left ventricular ejection fraction; LVESVI, left ventricular end-systolic volume index; LVEDVI, left ventricular end-diastolic volume index.

### The correlation of the plasma BNP levels with various clinical factors

Table 3 shows the Spearman rank correlation coefficients between the plasma BNP level and various clinical factors. The plasma BNP level was significantly and positively correlated with the age, FT4 level, TSH level, LVESVI, and LVEDVI (age: *r* = 0.349, *P* < 0.001; FT4: *r* = 0.206, *P* < 0.001; TSH: *r* = 0.124, *P* = 0.002; LVESVI: *r* = 0.445, *P* < 0.001; LVEDVI: *r* = 0.341, *P* < 0.001) and negatively correlated with the sex (male), BMI, DBP, Hb level, eGFR, FT3 level, and LVEF (sex: *r* = −0.216, *P* < 0.001; BMI: *r* = −0.168, *P* < 0.001; DBP: *r* = −0.204, *P* < 0.001; Hb: *r* = −0.298, *P* < 0.001; eGFR: *r* = −0.312, *P* < 0.001; FT3: *r* = −0.280, *P* < 0.001; LVEF: *r* = −0.420, *P* < 0.001).

**Table 3:** Spearman’s rank correlation coefficients between the BNP level and various clinical factors (n = 625).

	r	*P*
Male	− 0.216	< 0.001
Age	0.349	< 0.001
BMI	− 0.168	< 0.001
SBP	− 0.032	0.427
DBP	− 0.204	< 0.001
Heart rate	0.154	0.083
Hb	− 0.298	< 0.001
eGFR	− 0.312	< 0.001
FT3	− 0.280	< 0.001
FT4	0.206	< 0.001
TSH	0.124	0.002
LVEF	− 0.420	< 0.001
LVESVI	0.445	< 0.001
LVEDVI	0.341	< 0.001

BNP, B-type natriuretic peptide; BMI, body mass index; SBP, systolic blood pressure; DBP, diastolic blood pressure; Hb, hemoglobin; eGFR, estimated glomerular filtration rate; FT3, free triiodothyronine; FT4, free thyroxine; TSH, thyroid-stimulating hormone; LVEF, left ventricular ejection fraction; LVESVI, left ventricular end-systolic volume index; LVEDVI, left ventricular end-diastolic volume index.

### The correlation of the serum FT3 levels with various clinical factors

Table 4 shows the Spearman correlation coefficients between the serum FT3 level and various clinical factors. The serum FT3 level was significantly and positively correlated with the sex (male), BMI, DBP, Hb level, eGFR, and FT4 level (sex: *r* = 0.188, *P* < 0.001; BMI: *r* = 0.281, *P* < 0.001; DBP: *r* = 0.179, *P* < 0.001; Hb: *r* = 0.346, *P* < 0.001; eGFR: *r* = 0.197, *P* < 0.001; FT4: *r* = 0.091, *P* = 0.022) and negatively correlated with the age, TSH level, and BNP level (age: *r* = −0.290, *P* < 0.001; TSH: *r* = −0.160, *P* < 0.001; BNP: *r* = −0.280, *P* < 0.001), but LVEF, LVESVI, and LVEDVI were not significant.

**Table 4:** Spearman’s rank correlation coefficients between the FT3 level and various clinical factors (n = 625).

	r	*P*
Male	0.188	< 0.001
Age	− 0.290	< 0.001
BMI	0.281	< 0.001
SBP	0.000	0.996
DBP	0.179	< 0.001
Heart rate	− 0.071	0.429
Hb	0.346	< 0.001
eGFR	0.197	< 0.001
FT4	0.091	0.022
TSH	− 0.160	< 0.001
BNP	− 0.280	< 0.001
LVEF	0.060	0.136
LVESVI	− 0.050	0.215
LVEDVI	− 0.044	0.269

FT3, free triiodothyronine; BMI, body mass index; SBP, systolic blood pressure; DBP, diastolic blood pressure; Hb, hemoglobin; eGFR, estimated glomerular filtration rate; FT4, free thyroxine; TSH, thyroid-stimulating hormone; BNP, B-type natriuretic peptide; LVEF, left ventricular ejection fraction; LVESVI, left ventricular end-systolic volume index; LVEDVI, left ventricular end-diastolic volume index.

### The multiple regression analysis to determine the factors associated with the plasma BNP and serum FT3 levels in the whole group

Based on the statistical comparison of the two groups and results of a bivariate analysis, the multiple regression analysis for the BNP level included FT3, sex (male), age, BMI, DBP, Hb, eGFR, and LVEF as independent variables. As shown on Table 5, a multiple regression analysis revealed that LVEF, Hb, sex (male), FT3, and eGFR were significantly negatively, and age was positively associated with the log BNP (sex: *P* = 0.002, FT3 = 0.003, eGFR: *P* = 0.008, others: *P* < 0.001, respectively). In the same manner as the analysis for BNP, a multiple regression analysis for the FT3 level included BNP, sex (male), age, BMI, DBP, Hb, eGFR, and LVEF as independent variables. As shown on Table 6, a multiple regression analysis revealed that the log BNP and age were significantly negatively associated with the FT3 level, while the Hb value and BMI were positively associated with the FT3 level (age: *P* = 0.015, others: *P* < 0.001, respectively).

**Table 5:** Results of the multiple regression analysis to identify the clinical factors influencing log BNP (n = 625).

R^2^ = 0.436	Nonstandard coefficient		Standard regression coefficients	t value	*P* value	95% CI	VIF
	Regression coefficient	SE					
LVEF	− 0.050	0.003	− 0.493	− 15.795	< 0.001	− 0.056 to − 0.043	1.064
Age	0.025	0.004	0.219	6.139	< 0.001	0.017 to 0.032	1.386
Hb	− 0.114	0.026	− 0.155	− 4.341	< 0.001	− 0.166 to − 0.062	1.388
Male	− 0.387	0.121	− 0.103	− 3.185	0.002	− 0.625 to − 0.148	1.149
FT3	− 0.395	0.132	− 0.099	− 2.985	0.003	− 0.654 to − 0.135	1.196
eGFR	− 0.007	0.003	− 0.089	− 2.664	0.008	− 0.012 to − 0.002	1.215

BNP, B-type natriuretic peptide; R^2^, adjusted coefficient of determination; CI, confidence interval; VIF, variance inflation factor; LVEF, left ventricular ejection fraction; Hb, hemoglobin; FT3, free triiodothyronine; eGFR, estimated glomerular filtration rate.

**Table 6:** Results of the multiple regression analysis to identify the clinical factors influencing FT3 (n = 625).

R^2^ = 0.190	Nonstandard coefficient		Standard regression coefficients	t value	*P* value	95% CI	VIF
	Regression coefficient	SE					
Hb	0.038	0.008	0.209	5.072	< 0.001	0.024 to 0.053	1.285
Log BNP	− 0.043	0.010	− 0.172	− 4.386	< 0.001	− .0.062 to − 0.024	1.175
BMI	0.013	0.003	0.158	4.114	< 0.001	0.007 to 0.020	1.127
Age	− 0.003	0.001	− 0.100	− 2.436	0.015	− 0.005 to − 0.001	1.292

FT3, free triiodothyronine; R^2^, adjusted coefficient of determination; CI, confidence interval; VIF, variance inflation factor; Hb, hemoglobin; BNP, B-type natriuretic peptide; BMI, body mass index.

### The concept and results of the proposed path model (A) in the whole group

**Figure 1:**
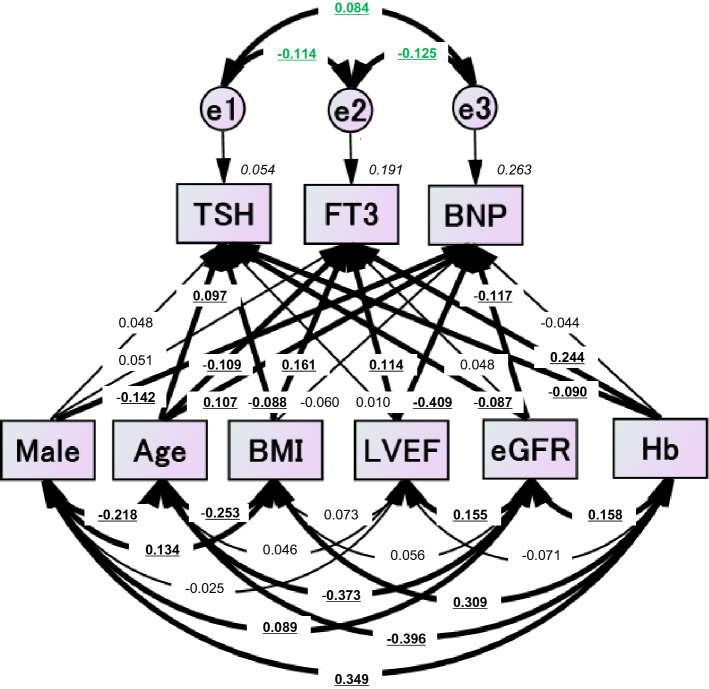
Path model (A) (the whole BNP group). The path model theoretically proposed to clarify the contribution of sex (male), age, BMI, LVEF, eGFR, or Hb to TSH, FT3 or BNP. The analysis was performed on all cases. Each path has a coefficient representing the standardized coefficient of a regressing independent variable on a dependent variable of the relevant path. These variables represent the standardized regression coefficients (direct effect) and squared multiple correlations (in narrow italics) as well as the correlations among exogenous variables (green). BMI, body mass index; LVEF, left ventricular ejection fraction; eGFR, estimated glomerular filtration rate; Hb, hemoglobin; TSH, thyroid-stimulating hormone; FT3, free triiodothyronine; BNP, B-type natriuretic peptide.

Based on multiple linear regression analyses and the findings of previous studies^29–32^, we selected sex (male), age, BMI, LVEF, eGFR, and Hb as independent variables and entered them into the path model. The theoretical path model (A) (Fig. 1) was proposed by positioning TSH, FT3, and BNP in parallel. The association between two factors was linked by two-way arrows. The sex (male), age, BMI, LVEF, eGFR, and Hb were placed in parallel, following arrows to TSH, FT3, and BNP, respectively. Paths were drawn from independent to dependent variables, with directional arrows for each regression model. As shown in Fig. 1 and Supplementary Table S1, path model (A) revealed that the age, BMI, LVEF, and Hb were associated with FT3 (age: standardized regression coefficient [*St. β*] = −0.109, *P* = 0.011, BMI: *St. β* = 0.161*, P* < 0.001*,* LVEF: *St. β* = 0.114, *P* = 0.002, Hb: *St. β* = 0.244, *P* =  < 0.001). Path model (A) also revealed that sex (male), age, LVEF, and eGFR were associated with BNP (male: *St. β* = −0.142, *P* < 0.001, age: *St. β* = 0.107, *P* = 0.009, LVEF: *St. β* = −0.409, *P* < 0.001, eGFR: *St. β* = −0.117, *P* = 0.002)*.* In addition, path model (A) showed an inverse association between FT3 and BNP (*St. β* = −0.125 *P* = 0.002), between TSH and FT3 (*St. β* = −0.114, *P* = 0.005), and between TSH and BNP (*St. β* = 0.084, *P* = 0.036).

### The relationship between BNP and FT3 in the normal BNP level group

**Figure 2:**
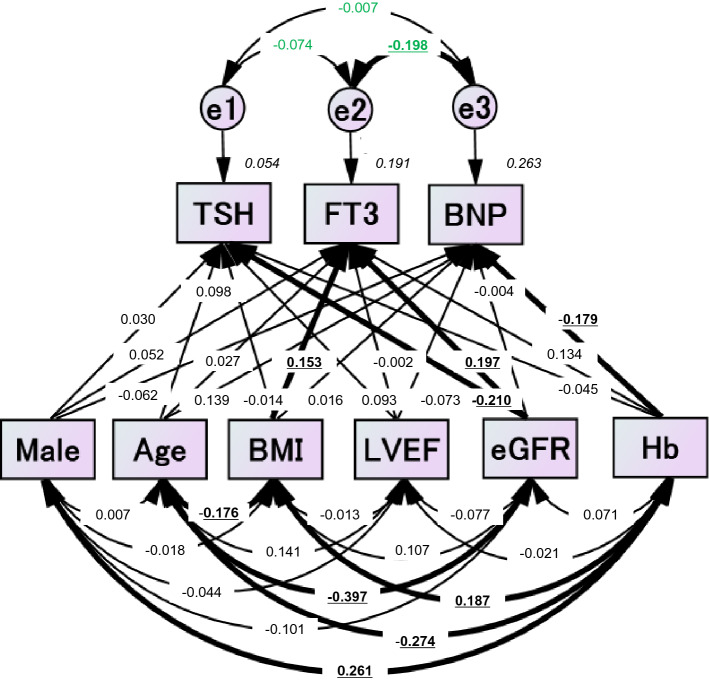
Path model (B) (the normal BNP level group). The path model theoretically proposed to clarify the contribution of sex (male), age, BMI, LVEF, eGFR, or Hb to TSH, FT3 or BNP. The analysis was performed only on patients in whom the BNP levels were within the normal range (BNP ≤ 18.4 pg/mL). Each path has a coefficient representing the standardized coefficient of a regressing independent variable on a dependent variable of the relevant path. These variables represent the standardized regression coefficients (direct effect) and squared multiple correlations (in narrow italics) as well as the correlations among exogenous variables (green). BMI, body mass index; LVEF, left ventricular ejection fraction; eGFR, estimated glomerular filtration rate; Hb, hemoglobin; TSH, thyroid-stimulating hormone; FT3, free triiodothyronine; BNP, B-type natriuretic peptide.

We defined the patient group that demonstrated BNP levels within the normal range as the “normal BNP level group”, and additional analyses were performed in the normal BNP level group (n = 185). As shown in Fig. 2 and Supplementary Table S2, path model (B) revealed that the BMI and eGFR were significantly associated with FT3 (BMI: *St. β* = 0.153, *P* = 0.034, eGFR: *St. β* = 0.197, *P* = 0.010), but LVEF was not significantly associated with FT3 (*St. β* = −0.002, *P* = 0.978). Path model (B) also revealed that the Hb was the only independent variable associated with BNP (*St. β* = −0.179, *P* = 0.022)*.* In addition, path model (B) showed no significant association between TSH and FT3 or between TSH and BNP but retained a significant inverse association between FT3 and BNP (*St. β* = −0.198, *P* = 0.008).

## Discussion

The present study showed that a decrease in the LVEF was significantly associated with a low serum T3 level, and there was a significant relationship between high BNP levels and low serum FT3 levels. Of note, in the normal BNP level group, the relationship between a high BNP level and low serum T3 level remained significant, while the LVEF was not significantly correlated with the serum FT3 level. However, whether a high BNP level led to the development of a low T3 level or vice versa remains unclear.

In the present study, a low FT3 level was significantly associated with an increase in the BNP level in patients with heart failure. Some previous reports have shown that a decrease in T3 is significantly related to an increase in BNP in patients with heart failure^23–26^. In humans, peripheral thyroid hormone metabolism is regulated by three iodothyronine deiodinases: D1, D2, and D3. D3 is present in the brain, skin, placenta, pregnant uterus, and various fetal tissues and catalyzes the conversion of T4 to reverse T3 (rT3) and the conversion of T3 to 3,3'-diiodothyronine (3,3'-T2), both of which are biologically inactive. Under pathological conditions, such as cancer, chronic inflammation, and critical illness, D3 is activated, the conversion of T3 to 3,3`-T2 is accelerated, and the serum T3 level consequently decreases^33–35^. In particular, the remarkable activation of D3 and a decrease in the serum T3 level are observed in patients with cardiovascular disorders^34,36,37^. However, in the present study, low FT3 levels associated with an increase in BNP were observed even in patients with normal BNP levels, thus indicating that low T3 is associated with high plasma BNP levels rather than worsening of hemodynamics. Perhaps a low T3 level might accelerate an increase in the BNP level. An unknown factor related to low T3 (e.g. thyroid hormone metabolites) might cause an increase in the BNP level. Further experiments will be needed to clarify what this unknown factor is.

In addition, Pinelli et al. showed an inverse correlation between FT3 and log NT-proBNP in non-cardiac patients, but it was a small-sample study (n = 52)^38^. In our study, 625 patients were involved, and high BNP levels were inversely correlated with low serum FT3 levels in patients with heart failure according to structural equation models (SEM), a relationship that was retained even in cases with normal BNP levels.

Several limitations associated with the present study warrant mention. First, this study was a retrospective one conducted at a single university hospital. Second, this study included patients with various cardiovascular diseases, and not all patients had apparent structural heart disease. Some patients in Stage A according to the Guidelines of Heart Failure^39,40^ were also included. Third, in the present study, the cardiac function of most patients was preserved, and the number of patients with heart failure with mid-range ejection fraction (HFmrEF) (n = 76) or heart failure with reduced ejection fraction (HFrEF) (n = 74) was small. Therefore, it was difficult to analyze the relationship between BNP and FT3 levels accurately in patients with HFmrEF or HFrEF. Based on an analysis of this current dataset, although the results were feasible in the patients with a preserved cardiac function, the results cannot be applied to those with HFmrEF or HFrEF. Finally, although we briefly checked the cardiac function of each patient by echocardiography before cardiac catheterization, we did not regularly register the precise echocardiography data and were thus unable to analyze the echocardiography data in detail in the present study.

In conclusion, there was a significant relationship between high plasma BNP levels and low serum FT3 levels in patients with heart failure, and this relationship was maintained even in cases with normal BNP levels. These findings indicate that low T3 is associated with high plasma BNP levels rather than worsening of hemodynamics.

## Methods

### Patient population

The study population consisted of 712 patients who were consecutively admitted to our institution with cardiovascular disorders and who underwent elective cardiac catheterization from September 2017 to February 2021. We excluded patients who underwent dialysis (n = 60). In addition, we excluded patients with already-known thyroid disorders and/or who were treated with thyroid medications (n = 27). Finally, we analyzed 625 patients in the present study (Supplementary Figure).

This study was conducted in accordance with the principles expressed in the Declaration of Helsinki and approved by the medical ethics committee of Jikei University School of Medicine [24–355(7121)]. All methods were carried out in accordance with relevant guideline and regulations. The Ethics Committee waived the need for informed written consent, since it was a retrospective study. Instead of obtaining informed consent from each patient, we posted a notice about the study design and contact information at a public location in our institution according to our routine ethical regulations.

### Data collection

The clinical characteristics of patients were retrospectively collected from their medical records. Blood samples were collected just before cardiac catheterization with the patient in a fasting condition. The LVEF, LVESVI, and LVEDVI were measured at the time of left ventriculography. Biochemical analyses of the plasma and serum were performed in our hospital’s central laboratory during the study period. To measure the plasma BNP level, blood samples were collected in tubes containing ethylenediaminetetraacetic acid (EDTA) and then immediately centrifuged at 3000 rpm for 5 min at 14 °C. Thereafter, the plasma BNP levels were immediately measured by a chemiluminescent enzyme immunoassay (CLEIA) with an AIA-CL2400 (TOSOH Corporation, Tokyo, Japan) as described in the previous report^41^. The normal range of plasma BNP level was set at ≤ 18.4 pg/mL. The serum FT3, FT4, and TSH levels were measured by the CLEIA method using a CL AIA-PACK FT3 TEST CUP, CL AIA-PACK FT4 TEST CUP, and CL AIA-PACK TSH TEST CUP, respectively (TOSOH Corporation).

### Statistical analyses

Data are expressed as the mean ± standard deviation (SD) or as the median (25th, 75th percentile) for significantly skewed variables. For continuous variables, differences between the two groups were evaluated either by an unpaired Student’s t-test or the Mann -Whitney rank-sum test. For discrete variables, which were expressed as counts and percentages, any differences between the two groups were analyzed by the chi-square test, unless the expected values in any cells were less than 5, in which case Fisher’s exact test was used. Correlations between the clinical parameters and the BNP or FT3 levels were assessed using Spearman’s rank correlation coefficient. First, to exclude the effect of other factors in clarifying the relationship between BNP and FT3, a step-wise multiple linear regression analysis was performed with BNP as the dependent variable. The independent variables were selected based on theoretical grounds, the results of the statistical comparison of two groups and a bivariate analysis. Similarly, a step-wise multiple regression analysis was repeated with FT3 as the dependent variable using the same independent variables. All statistical analyses were performed using the SPSS Statistics software program (version 27.0; SPSS Inc., Chicago, IL, USA). *P* values of < 0.05 were considered to indicate statistical significance.

In addition, a path model based on a SEM was used to investigate the relationships between the BNP and FT3 levels. The path model defined some hierarchical regression models among clinical factors and the BNP and FT3 levels. A path analysis was performed using the IBM SPSS AMOS software program (version 27; Amos Development Corporation, Meadville, PA, USA). The SEM that were obtained were tested and confirmed at a significance level of *P* < 0.05.

The following points should be noted regarding SEM: First, SEM is an effective analytical method for confirming hypotheses, but analysts need to consider sufficient hypotheses before and after building a path model. By maximizing the analyst's knowledge, the hypothesis approaches the appropriate model. In other words, it takes trial and error to create a proper path diagram. Second, "causality" is an issue that needs to be addressed. Strictly speaking, in order to build a causal relationship, it is necessary to discuss the pre-occurrence priority in terms of the temporal priority in which the causal relationship occurs. Care must be taken to conclude that there was an exact causal relationship without such consideration. If the temporal relationship is unclear, it should simply be called a relationship. In the present study, we planned to devise an optimal path diagram in consideration of these points. However, we may need to look at other path diagrams in the future.

## Supplementary Information

Supplementary Information.
