# Supplementary material for: Low triiodothyronine levels correlate with high B-type natriuretic peptide levels in patients with heart failure
Source: Sci Rep. 2021 Nov 8;11:21865. doi: 10.1038/s41598-021-01454-5 (PMC8576007; doi:10.1038/s41598-021-01454-5)

**Supplementary Information**

**Low Triiodothyronine Levels Correlate with High B-type Natriuretic Peptide Levels in Patients with Heart Failure**

Hirotake Takahashi*^1^, Yusuke Kashiwagi*^1^, Tomohisa Nagoshi^1^, Yoshiro Tanaka^1^, Yuhei Oi^1^, Haruka Kimura^1^, Kousuke Minai^1^, Michihiro Yoshimura^1^

^1^ Division of Cardiology, Department of Internal Medicine, The Jikei University School of Medicine, Tokyo, Japan

* Hirotake Takahashi and Yusuke Kashiwagi contributed equally to this article.

**Supplementary Table S1. Results of path model A (the whole group) (n=625).**

| Clinical factors | | | Estimate | Standard error | Test statistic | *P* value |
| --- | --- | --- | --- | --- | --- | --- |
| TSH | <--- | Male | 0.203 | 0.176 | 1.152 | 0.249 |
|  | <--- | Age | 0.012 | 0.006 | 2.098 | 0.036 |
|  | <--- | BMI | -0.033 | 0.016 | -2.101 | 0.036 |
|  | <--- | LVEF | 0.001 | 0.005 | 0.260 | 0.795 |
|  | <--- | eGFR | -0.007 | 0.004 | -2.023 | 0.043 |
|  | <--- | Hb | -0.075 | 0.038 | -1.970 | 0.049 |
| FT3 | <--- | Male | 0.049 | 0.037 | 1.327 | 0.184 |
|  | <--- | Age | -0.003 | 0.001 | -2.557 | 0.011 |
|  | <--- | BMI | 0.014 | 0.003 | 4.180 | <0.001 |
|  | <--- | LVEF | 0.003 | 0.001 | 3.077 | 0.002 |
|  | <--- | eGFR | 0.001 | 0.001 | 1.205 | 0.228 |
|  | <--- | Hb | 0.046 | 0.008 | 5.776 | <0.001 |
| BNP | <--- | Male | -62.786 | 16.258 | -3.862 | <0.001 |
|  | <--- | Age | 1.403 | 0.535 | 2.621 | 0.009 |
|  | <--- | BMI | -2.343 | 1.431 | -1.637 | 0.102 |
|  | <--- | LVEF | -4.818 | 0.416 | -11.588 | <0.001 |
|  | <--- | eGFR | -1.036 | 0.336 | -3.086 | 0.002 |
|  | <--- | Hb | -3.795 | 3.492 | -1.087 | 0.277 |
| Male | <--> | Age | -0.852 | 0.160 | -5.330 | <0.001 |
|  | <--> | BMI | 0.178 | 0.053 | 3.328 | <0.001 |
|  | <--> | LVEF | -0.109 | 0.175 | -0.625 | 0.532 |
|  | <--> | eGFR | 0.514 | 0.232 | 2.213 | 0.027 |
|  | <--> | Hb | 0.207 | 0.025 | 8.221 | <0.001 |
| Age | <--> | BMI | -11.194 | 1.829 | -6.119 | <0.001 |
|  | <--> | LVEF | 6.798 | 5.859 | 1.160 | 0.246 |
|  | <--> | Hb | -7.888 | 0.858 | -9.190 | <0.001 |
|  | <--> | eGFR | -72.179 | 8.276 | -8.721 | <0.001 |
| BMI | <--> | LVEF | 3.628 | 1.988 | 1.825 | 0.068 |
|  | <--> | eGFR | 3.707 | 2.631 | 1.409 | 0.159 |
|  | <--> | Hb | 2.081 | 0.283 | 7.356 | <0.001 |
| LVEF | <--> | eGFR | 33.551 | 8.771 | 3.825 | <0.001 |
|  | <--> | Hb | -1.584 | 0.894 | -1.772 | 0.076 |
| GFR | <--> | Hb | 4.667 | 1.197 | 3.900 | <0.001 |
| e1 | <--> | e2 | -0.046 | 0.016 | -2.817 | 0.005 |
|  | <--> | e3 | 15.246 | 7.267 | 2.098 | 0.036 |
| e2 | <--> | e3 | -4.733 | 1.522 | -3.109 | 0.002 |

TSH, thyroid-stimulating hormone; BMI, body mass index; LVEF, left ventricular ejection fraction; eGFR, estimated glomerular filtration rate; Hb, hemoglobin; FT3, free triiodothyronine; BNP, B-type natriuretic peptide.

**Supplementary Table S2. Results of path model B** **(the normal BNP level group) (n=185).**

| Clinical factors | | | Estimate | Standard error | Test statistic | *P* value |
| --- | --- | --- | --- | --- | --- | --- |
| TSH | <--- | Male | 0.170 | 0.420 | 0.404 | 0.686 |
|  | <--- | Age | 0.011 | 0.009 | 1.213 | 0.225 |
|  | <--- | BMI | -0.005 | 0.024 | -0.200 | 0.841 |
|  | <--- | LVEF | 0.016 | 0.012 | 1.310 | 0.190 |
|  | <--- | eGFR | -0.016 | 0.006 | -2.723 | 0.006 |
|  | <--- | Hb | -0.042 | 0.073 | -0.578 | 0.563 |
| FT3 | <--- | Male | 0.063 | 0.089 | 0.705 | 0.481 |
|  | <--- | Age | 0.001 | 0.002 | 0.331 | 0.740 |
|  | <--- | BMI | 0.011 | 0.005 | 2.119 | 0.034 |
|  | <--- | LVEF | 0.000 | 0.003 | -0.028 | 0.978 |
|  | <--- | eGFR | 0.003 | 0.001 | 2.564 | 0.010 |
|  | <--- | Hb | 0.027 | 0.016 | 1.741 | 0.082 |
| BNP | <--- | Male | -1.411 | 1.691 | -0.834 | 0.404 |
|  | <--- | Age | 0.061 | 0.036 | 1.705 | 0.088 |
|  | <--- | BMI | 0.022 | 0.098 | 0.222 | 0.824 |
|  | <--- | LVEF | -0.049 | 0.048 | -1.013 | 0.311 |
|  | <--- | eGFR | -0.001 | 0.024 | -0.053 | 0.958 |
|  | <--- | Hb | -0.677 | 0.296 | -2.289 | 0.022 |
| Male | <--> | Age | 0.017 | 0.176 | 0.095 | 0.925 |
|  | <--> | BMI | -0.014 | 0.058 | -0.238 | 0.812 |
|  | <--> | LVEF | -0.069 | 0.116 | -0.594 | 0.553 |
|  | <--> | eGFR | -0.343 | 0.251 | -1.368 | 0.171 |
|  | <--> | Hb | 0.072 | 0.021 | 3.410 | <0.001 |
| Age | <--> | BMI | -7.117 | 3.030 | -2.349 | 0.019 |
|  | <--> | LVEF | 11.446 | 6.045 | 1.894 | 0.058 |
|  | <--> | eGFR | -69.435 | 13.880 | -5.003 | <0.001 |
|  | <--> | Hb | -3.933 | 1.102 | -3.568 | <0.001 |
| BMI | <--> | LVEF | -0.352 | 1.959 | -0.180 | 0.857 |
|  | <--> | eGFR | 6.150 | 4.247 | 1.448 | 0.148 |
|  | <--> | Hb | 0.877 | 0.354 | 2.478 | 0.013 |
| LVEF | <--> | eGFR | -8.869 | 8.494 | -1.044 | 0.296 |
|  | <--> | Hb | -0.200 | 0.698 | -0.286 | 0.775 |
| eGFR | <--> | Hb | 1.447 | 1.508 | 0.959 | 0.337 |
| e1 | <--> | e2 | -0.022 | 0.021 | -1.001 | 0.317 |
|  | <--> | e3 | -0.039 | 0.406 | -0.097 | 0.923 |
| e2 | <--> | e3 | -0.232 | 0.088 | -2.637 | 0.008 |

BNP, B-type natriuretic peptide; TSH, thyroid-stimulating hormone; BMI, body mass index; LVEF, left ventricular ejection fraction; eGFR, estimated glomerular filtration rate; Hb, hemoglobin; FT3, free triiodothyronine.

**Supplemental Table S3. Detailed description of underlying main cardiovascular disease**

| **Ischemic heart disease (n)** | 506 |
| --- | --- |
| stable AP (n) | 300 |
| unstable AP (n) | 22 |
| OMI (n) | 131 |
| SMI (n) | 89 |
| CSA (n) | 17 |
| **Valvular disease (n)** | 18 |
| AS (n) | 4 |
| AR (n) | 5 |
| MR (n) | 9 |
| TR (n) | 3 |
| MS (n) | 2 |
| **Arrhythmia (n)** | 10 |
| Af (n) | 7 |
| AFL (n) | 1 |
| VF (n) | 1 |
| AVB (n) | 1 |
| SSS (n) | 2 |
| **Macrovascular disease (n)** | 2 |
| Preoperative TAA (n) | 2 |
| **Congenital heart disease (n)** | 1 |
| Preoperative ASD (n) | 1 |

AP, angina pectoris; OMI, old myocardial infarction; SMI, silent myocardial ischemia; CSA, coronary spastic angina, AS, aortic valve stenosis; AR, aortic valve regurgitation; MR, mitral valve regurgitation; MS, mitral valve stenosis; TR, tricuspid valve regurgitation; Af, atrial fibrillation; AFL, atrial flutter; VF, ventricular fibrillation; AVB, atrioventricular block; SSS, sick sinus syndrome. (Some patients overlapped multiple disease, for example: MR and TR)

**Supplemental Table S4. Classification of patients with heart failure according to LVEF (n=608).**

| **Based on LVEF (n)** |  |
| --- | --- |
| HFpEF (≥50%) (n) | 458 |
| HFmrEF (40-49%) (n) | 76 |
| HFrEF (<40%) (n) | 74 |

LVEF, left ventricular ejection fraction; HF, heart failure.

**Supplemental Table S5. BNP level of each ACCF/AHA Stage of heart failure (n=625).**

| **ACCF/AHA Stage of heart failure** | **The median (25th, 75th percentile) BNP level (pg/mL)** |
| --- | --- |
| Stage A | 25.8 (10, 46) |
| Stage B | 26.7 (13.9, 60.4) |
| Stage C | 139 (50.9, 245) |
| Stage D | 832 (412, 1644) |

BNP, B-type natriuretic peptide; ACCF, American College of Cardiology Foundation; AHA, American Heart Association.

**Supplementary Table S6. A comparison of the clinical characteristics between the normal BNP level group and the raised BNP level group.**

| Characteristics | **normal BNP level group (n=185)** | **raised BNP level group (n=440)** | ***P* value** |
| --- | --- | --- | --- |
| Male (n, %) | 176 (95.1) | 365 (83.0) | <0.001 |
| Age (years) | 60 (52, 69) | 69 (61, 76) | <0.001 |
| BMI (kg/m^2^) | 24.5 (22.9, 26.7) | 24.0 (21.8, 26.4) | 0.006 |
| SBP (mmHg) | 133 (114, 147) | 134 (116, 152) | 0.368 |
| DBP (mmHg) | 72 (66, 83) | 69 (61, 76) | <0.001 |
| Heart rate (beats per minutes) | 63 (60, 76) | 74 (61, 85) | 0.135 |
| Hb (g/dL) | 14.6 ± 1.29 | 13.7 ± 1.83 | <0.001 |
| eGFR (mL/min/1.73 m^2^) | 75.0 (67.6, 86.7) | 69.0 (60.4, 81.8) | <0.001 |
| Total bilirubin (mg/dL) | 0.8 (0.6, 0.9) | 0.8 (0.6, 1.0) | 0.612 |
| Albumin (g/dL) | 4.16 ± 0.29 | 3.84 ± 0.38 | <0.001 |
| Fasting blood sugar (mg/dL) | 104 (94, 123) | 105 (94, 126) | 0.855 |
| HbA1c (%) | 6.0 (5.6, 6.7) | 6.1 (5.7, 6.7) | 0.200 |
| HDL (mg/dL) | 50 (41, 61) | 49 (41, 60) | 0.699 |
| LDL (mg/dL) | 93 (79, 107) | 94 (74, 114) | 0.914 |
| Triglyceride (mg/dL) | 107 (81, 149) | 97 (71, 132) | 0.015 |
| FT3 (pg/mL) | 2.42 ± 0.26 | 2.29 ± 0.34 | <0.001 |
| FT4 (ng/dL) | 1.20 ± 0.20 | 1.27 ± 0.24 | <0.001 |
| TSH (μIU/mL) | 1.26 (0.76, 2.05) | 1.48 (0.96, 2.30) | 0.013 |
| LVEF (%) | 63.3 (59.9, 66.9) | 59.0 (46.9, 65.4) | <0.001 |
| LVESVI (mL/m^2^) | 20.1 (16.5, 25.2) | 25.3 (18.9, 38.5) | <0.001 |
| LVEDVI (mL/m^2^) | 57.3 (48.3, 67.8) | 63.3 (51.6, 78.7) | <0.001 |
| Underlying main cardiovascular disease (n, %) |  |  |  |
| Ischemic heart disease | 167 (90.2) | 339 (77.0) | <0.001 |
| Cardiomyopathy | 2 (1.1) | 50 (11.4) | <0.001 |
| Valvular disease | 0 (0) | 18 (4.1) | 0.005 |
| Arrhythmia | 2 (1.1) | 8 (1.8) | 0.392 |
| Macrovascular disease | 1 (0.5) | 1 (0.2) | 0.505 |
| Congenital heart disease | 0 (0) | 1 (0.2) | 0.704 |
| Other diseases | 13 (7.0) | 23 (5.2) | 0.378 |
| Medications (n, %) |  |  |  |
| Antiplatelet agent | 165 (89.2) | 326 (74.1) | <0.001 |
| ACE inhibitor | 48 (25.9) | 137 (31.1) | 0.194 |
| ARB | 74 (40.0) | 171 (38.9) | 0.791 |
| Beta-blocker | 64 (34.6) | 256 (58.2) | <0.001 |
| Diuretics | 14 (7.6) | 135 (30.7) | <0.001 |

SD, standard deviation; BMI, body mass index; SBP, systolic blood pressure; DBP, diastolic blood pressure; Hb, hemoglobin; eGFR, estimated glomerular filtration rate; HDL, high-density lipoprotein cholesterol; LDL, low-density lipoprotein cholesterol; FT3, free triiodothyronine; FT4, free thyroxine; TSH, thyroid-stimulating hormone; BNP, B-type natriuretic peptide; LVEF, left ventricular ejection fraction; LVESVI, left ventricular end-systolic volume index; LVEDVI, left ventricular end-diastolic volume index; ACE, angiotensin-converting enzyme; ARB, angiotensin II receptor blocker.

Data are presented as the number (%), mean ± SD or median (25th, 75th percentile).

**Supplementary Figure Legend**

**Supplementary Figure:** **Patient flow chart for the present study.** We excluded 60 patients who underwent dialysis and 27 who had already-known thyroid disorders and/or were being treated with thyroid medications.


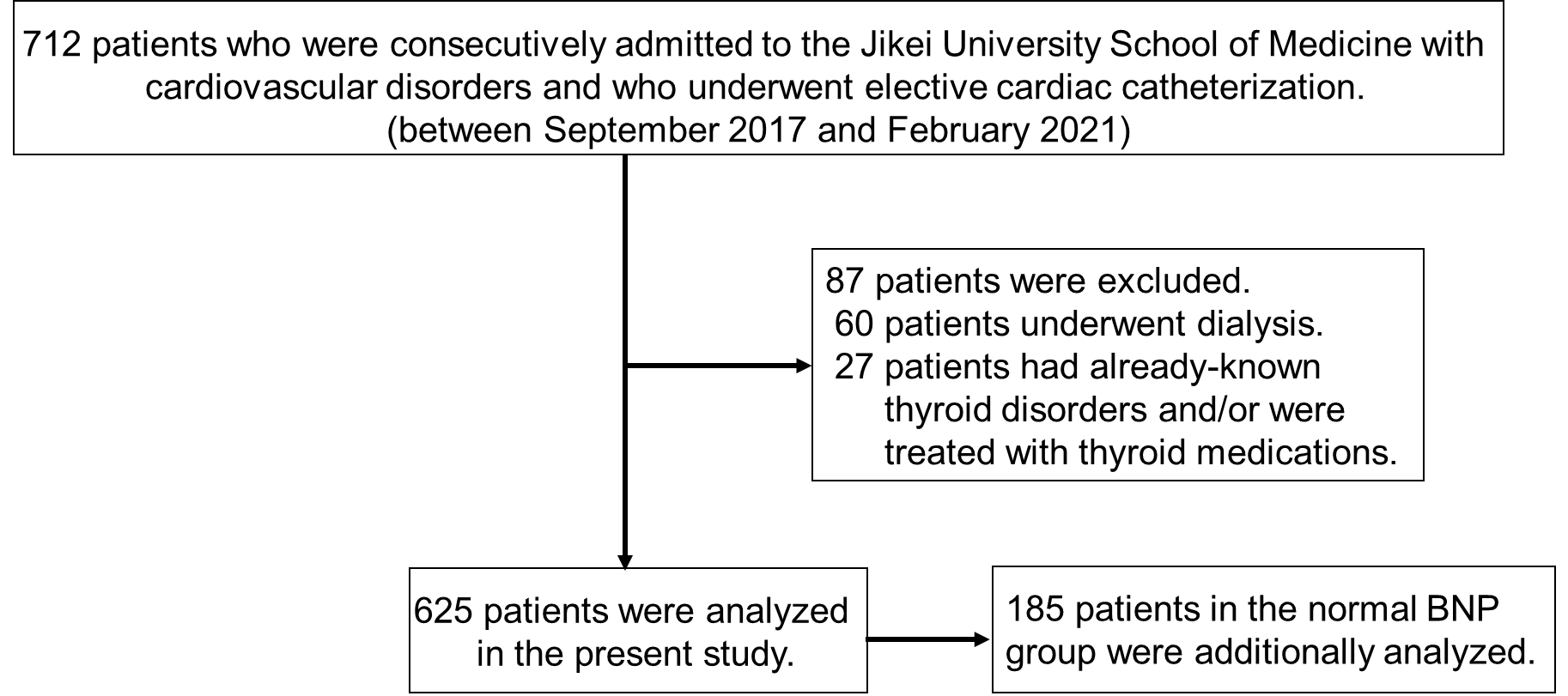

Supplement: Supplementary file 1 — Supplementary Information. [file 41598_2021_1454_MOESM1_ESM.docx]
